# Awareness and attitudes towards emergency contraceptive pills among young people in the entertainment places, Vientiane City, Lao PDR

**DOI:** 10.1186/1472-6874-13-14

**Published:** 2013-03-21

**Authors:** Vanphanom Sychareun, Visanou Hansana, Alongkone Phengsavanh, Keokedthong Phongsavan

**Affiliations:** 1 Faculty of Postgraduate Studies, University of Health Sciences, Ministry of Health, P.O. Box 7444, Vientiane, Lao PDR; 2 Obstetrics-Gynecology Section, Sethathirath Hospital, Ministry of Health, Vientiane, Lao PDR

## Abstract

**Background:**

Emergency Contraception is not officially available to the public sector in Laos. The potential of emergency contraception to prevent unwanted pregnancies is well documented in developed countries, but in Laos no studies of ECPs exist. This study aimed to assess knowledge of and attitudes towards emergency contraceptive pills (ECPs) in Vientiane, the capital city of the Lao PDR.

**Methods:**

A cross-sectional survey was conducted among 500 young adults in entertainment venues by using the convenience sampling between May to July, 2007. Data were obtained through face-to-face interview. Participants were asked about socio- demographic characteristics, knowledge, attitudes related to ECPs, and source of information about ECPs. Data analysis was performed with chi-square test and logistic regression (p < .05).

**Results:**

Only 22.4 percent of respondents had heard of ECPs and of these only 17.9 percent knew the correct time-frame for effective use. Most of the respondents (85%) agreed on the need for ECPs to be available in Laos and 66.8 percent stated that they would use them should the need arise, if they were available. Among those who said they would not use ECPs, 63.8 percent were concerned about possible health effects, or other side effects. Awareness of ECPs was associated with increasing age (OR = 2.78, p = .025) and male sex (OR = 2.91, p = .010).

**Conclusions:**

There is needed to provide effective health education about the method, timing of use, and how to obtain ECPs through both informal, peer channels, and also through formal channels such as health care providers.

## Background

Unintended pregnancy continues to be a significant public health issue and poses a major challenge to the reproductive health of women, and particularly among young adults in developing countries. Some teenagers with unintended pregnancies resort to abortions, which are often performed under unhygienic and potentially life threatening conditions and others bear their pregnancies to term, incurring the risk of morbidity and mortality related to pregnancy and delivery, together with serious social risks [1,2]. The World Health Organization estimated nearly 21,600,000 unsafe abortions took place in 2008, almost all in developing countries with restrictive abortion laws [3]. A significant proportion of maternal mortality worldwide is attributable to unsafe induced abortion, and this is the case in the Lao PDR. The country has a maternal mortality rate of around 405 per 100,000 live births and one of the major causes of maternal deaths is complications of unsafe abortions [4].

Lao PDR is a mountainous land locked country in South East Asia and has a population with a wide ranging socio-cultural and ethnic composition. It has a population of 5.6 million [5]. The Lao government has adopted many progressive family planning policies since 1993, though the fertility rate of the country is still high (Total Fertility Rate of 4.5) [4]. The prevalence of using any contraceptive method and any modern method among currently married women of reproductive age is 38.4 percent and 35 percent respectively. The most popular modern method is the oral contraceptive pill (15.9%), followed by injectable contraceptives (10.6%) and the intrauterine contraceptive device (2.9%) [5].

In the Lao PDR, abortion is highly restricted and in most cases illegal; the majority of procedures are clandestine, and a high proportion are unsafe. Abortion is allowed only to save the life of pregnant women; however, there is very limited data on the incidence of abortion.

Given the negative consequences of an unwanted pregnancy and the legal status of abortion in the Lao PDR, the use of emergency contraceptives is potentially very important. Emergency contraceptive pills (ECPs), also known as postcoital contraception, are most commonly based on hormonal regimens, including a combination of ethinyl estradiol with levonorgestrel (Yuzpe regimen) and levonorgestrel alone [6], Ulipristal acetate30 mg [7]. ECPs in the past were considered to be effective only within 72 hours after intercourse, but recent studies have confirmed that they are effective for up to 120 hours [8,9]. Their effectiveness has been claimed to be between 75–80 percent [10]. The fact that ECPs are not 100 percent effective needs to be disseminated to the providers as well as the women who may use the pills. The male partner should also be informed and educated about the advantages and disadvantages of ECPs.

However, ECPs are not included in the National Guidelines of the Family Planning Program in Laos even though the Ministry of Health endorsed a new “National Reproductive Health Policy” in 2005 [11] which approved providing emergency contraception to the health facilities in the country. No pharmaceutical product has been registered for emergency contraception in Laos yet; however, some ECPs are available over the counter at drug stores, imported illegally from neighboring countries. Potential users could therefore buy emergency contraception directly from some drugstores if they are aware of the drugs and the stores that carry supplies.

To date, no research on ECPs among young adults has been conducted in the country. Thus the aim of this study was to investigate the awareness and attitudes of young adults regarding emergency contraception and their willingness to use ECPs if they are introduced in the public and private sectors. It is hoped that this information will help to develop appropriate strategies to increase the availability and appropriate usage of the method within the family planning program and reduce the incidence of unwanted pregnancy and its consequences in Laos.

## Methods

### Study setting

The study was carried out in Vientiane, Lao PDR, which has a population of 700,000 and the highest population density in the country. Compared with other parts of the country, the city has both a higher level of education and income. Vientiane is the center of culture, commerce and administration in Laos [5].

### Study population and sampling

This study was carried out from May to July, 2007. A structured questionnaire was used to assess the knowledge and perceptions regarding ECPs among the respondents, as well as their willingness to use ECPs if required. The sample size was determined using single population proportion formula assuming; 95percent level of confidence, proportion of awareness of EC use of 50 percent, precision (4%) and non-response of 10 percent. This made the final sample size 550 adults with the ratio of female to male 4:1. The ratio of female to male is 4:1 because ECPs are used by women and the sole role of males is participation in decision making about the use of contraceptive methods.

The questionnaire was initially designed taking into consideration similar surveys that have been carried out in other countries [12]. We modified the original questionnaire to be appropriate to our context. The survey questionnaire had both closed and open-ended questions and consisted of four sections. The socio-demographic section included questions on age, gender, education, professional status, and marital and family status. The knowledge on Emergency Contraceptive Pills section consisted of questions about having heard about ECPs, source of information on ECPs (multiple responses), how ECPs are used (multiple responses), and the mode of action of ECPs. In addition, for those who did not know about ECPs, there was a question regarding what information they would like to know about ECPs with multiple responses permitted. The attitudes towards Emergency Contraception Pills section consisted of perceptions related to potential ECPs users, its availability, and willingness to use ECPs if needed. The attitudinal questions used a Likert scale ranging from (1) strongly disagree, (2) disagree, (3) agree and (4) strongly agree. Due to the paucity of responses on the “strongly disagree” and “strongly agree” resposne options, the categories of strongly disagree and disagree were combined together as “disagree” and similarly, categories of strongly agree and agree also combined together as “agree” for the analysis [13]. Items related to reasons of use and non use of ECPs and the most appropriate method to disseminate information on ECPs, if there will be disseminated of ECPs also included with multiple responses. The practice as regards ECPs section included whether respondents had ever used ECPs, and if so the frequency of use, method of use, side effects and sources of ECPs: however, there were only a few respondents who answered on having ever used ECPs, and therefore the responses regarding the ever use of ECPs were not included in the analysis.

The questionnaires were administered face-to-face at different entertainment venues such as bars, nightclubs and beer shops where young people from various sectors (school, university, workplace, etc.…) gathered. The list of entertainment places was prepared as the sampling frame, then 20 entertainment places were randomly selected from the constructed sampling framework. A request and appointment to visit for the purposes of recruitment was made with the manager or owner of the nightclubs before each visit. Respondents were approached as they entered in the nightclubs. A trained interviewer approached every other person entering to determine eligibility. Eligibility criteria were age 15–30 years, and being a client of the nightclubs. If the selected subject did not meet the inclusion criteria, the next person entering was selected. Recruitment continued until all interviewers were occupied. Once an interviewer became available, recruitment recommenced. Eligible subjects were invited to a private area set up by the study team inside or outside the entertainment establishment. The purpose of the study was explained to them, and they were then invited to participate in the study.

### Data collection

Data was collected by the research team from the Faculty of Postgraduate Studies, University of Health Sciences. Before data collection, six interviewers and one supervisor were trained regarding the purpose of the study and the details of the questionnaire. The questionnaire was pretested with 20 respondents in entertainment places and the responses were then studied to identify the major issues that needed modification, before finalizing the tool. The interview took approximately 20–30 min and included both closed- and open-ended questions. The interviewers were young men and women who could easily identify with the participants. Subjects were interviewed by researchers of their own sex.

### Data analysis

Data analysis was undertaken using SPSS (version10.0). Descriptive statistics were used to analyze the data. Frequency and percentage distribution were calculated for all items. Chi-square tests were used to determine differences between women and men. Logistic regression examined factors associated with awareness of ECPs by controlling for other confounders such as socio-demographic variables. Significance level was set at p-value less than .05. We also calculated the effect size which suggests how strong the relationship between the groups and provides information about the size or magnitude of the effect [14].

This study was approved by the National Ethical Committee Review Board, Ministry of Health, Lao PDR. Informed consent was obtained verbally and participation was anonymous. No names or other personally identifying information were recorded. Subjects aged 15–18 years were considered emancipated minors and able to consent to the study.

## Results

### Socio-demographic characteristics

A total of 500 young adults were interviewed with a response rate of 91 percent. The main reason given for not participating in the interview was lack of time. The main characteristics of the demographic profile are shown in the Table 1. Four hundred female and one hundred male adults in Vientiane participated in the survey with the mean age of the respondents being 21.7 ± 3.7 (range of 15 to 30 years). The majority of participants had finished senior secondary school (42.4%); 25.8% had a bachelor degree or higher. Less than half of the respondents (44.2%) were students while 25.4 percent were public servants or worked in the private sector. Most (86.8%) were single. There was a difference according to sex in education and occupation: males reported a higher level of education than females (p < .005), and this was reflected in their occupation. However, the strength of the relationship between the male and female groups in education and occupation is small.

**Table 1:** Socio-demographic characteristics of respondents (n = 500)

**Characteristics**	**Male**		**Female**		**Total**		**Chi-square**	**P-value**	**Effect size**
	**N = 100**	**%**	**N = 400**	**%**	**N = 500**	**%**			
**Age**							4.908	0.086	0.099
15-19	31	31.0	140	35.0	171	34.2			
20-25	52	52.0	160	40.0	215	43.0			
26- 30	17	17.0	97	24.3	114	22.8			
**Marital status**							4.338	0.114	0.093
Single	93	93.0	341	85.3	434	86.8			
Married	7	7.0	57	14.3	64	12.8			
Divorced	0	0.0	2	0.5	2	0.4			
**Education**							18.128	0.003	0.190
Primary	1	1.0	20	5.0	21	4.2			
Junior Secondary	3	3.0	52	13.0	55	11.0			
Senior Secondary	55	55.0	157	39.3	212	42.4			
Vocational	2	2.0	26	6.5	28	5.6			
Technician	11	11.0	44	11.0	55	11.0			
Bachelor degree & other	28	28.0	101	25.3	129	25.8			
**Occupation**							14.62	0.023	0.171
Student	58	58.0	163	40.8	221	44.2			
Public servant	16	16.0	111	27.8	127	25.4			
Laborer	8	8.0	41	10.3	49	9.8			
Merchants / Business	9	9.0	42	10.5	51	10.2			
Unemployed	8	8.0	23	5.8	31	6.2			
House wife	0	0.0	14	3.5	14	2.8			
Farmer	1	1.0	6	1.5	7	1.4			
**Religion**							9.217	0.009	0.135
Buddhist	85	85.0	376	94.0	461	92.2			
Animist	13	13.0	20	5.0	33	6.6			
Christian	2	2.0	4	1.0	6	1.2			

Note: .10 - small effect size.

30 - medium effect size.

50 - large effect size.

### Knowledge about Emergency Contraceptive Pills (ECPs)

One hundred and twelve respondents (22.4%) reported that they had heard about emergency contraception. There was no significant difference between men and women regarding knowledge of ECPs (24.0% vs 22.0%, p > .05). Among those aware of ECPs, 50 percent said they received their information from friends, 24.1 percent heard about it from the media and health personnel for each and 21.4 percent obtained it from their mothers or sisters (Table 2).

**Table 2:** Respondents who had heard of ECPs and their source of information (n = 500)

**Variables**	**Male**		**Female**		**Total**		**Chi-square**	**P-value**	**Effect size**
	**n = 100**	**%**	**n = 400**	**%**	**N = 500**	**%**			
**Have Heard of ECPs**									
Yes	24	24.0	88	22.0	112	22.4	0.184	0.668	0.019
No	76	76.0	312	78.0	388	77.6			
**Sources of information about ECPs (n = 122)**									
	**Male**		**Female**		**Total**				
	**N = 24**	**%**	**N = 88**	**%**	**N = 112**	**%**			
From friends									
Yes	14	58.3	42	47.7	56	50	1.859	0.357	0.123
No	10	41.7	46	52.3	56	50			
From health personnel									
Yes	5	20.8	22	25.0	27	24.1	0.178	0.672	0.040
No	19	79.2	66	75.0	85	75.9			
From media ( Radio, TV, magazine, etc.)									
Yes	5	20.8	22	25.0	27	24.1	0.178	0.672	0.040
No	19	79.2	66	75.0	85	75.9			
From mother or sister									
Yes	2	8.3	22	25.0	24	21.4	3.11	0.078	0.166
No	22	91.7	66	75.0	88	78.6			
From pharmacy									
Yes	3	12.5	8	9.1	11	9.8	.247	0.700	0.047
No	21	87.5	80	90.9	101	90.2			

Note: .10 - small effect size.

.30 - medium effect size.

.50 - large effect size.

While 89.3 percent believed that ECPs were effective in preventing pregnancy, 95.5 percent recognized that they could not prevent the spread of HIV. Women had a lower level of belief in the effectiveness of ECPs in preventing a pregnancy (88.6% vs 91.7%; p > .05) in comparison with men (Table 3). Women had a greater recognition of the inability of ECPs to prevent HIV (97.7% vs 87.5%; p > .059). About one third of respondents (35.7%) knew that the drug was not an abortion pill and there was no difference between males and females (38.6% vs 25%, p > .05).

**Table 3:** Knowledge about Emergency Contraception Pills by those who had ever heard about them (n = 112) with yes answers

**Variables**	**Male**		**Female**		**Total**		**Chi-square**	**P-value**	**Effect size**
	**n = 24**	**%**	**N = 88**	**%**	**N = 112**	**%**			
**Knowledge of benefit using ECPs**									
Can prevent pregnancy	22	91.7	78	88.6	100	89.3	.0.181	.0.671	0.040
Can prevent HIV/AIDS	3	12.5	2	2.3	5	4.5	4.626	0.032	0.203
Can cause abortion	6	25.0	34	38.6	40	35.7	1.527	0.217	0.116
**Knowledge of usage ECPs**									
Take one contraception pill per day	8	33.3	23	26.1	31	27.7	0.487	0.48.5	0.066
Take contraception pill after sexual intercourse within 72 hrs	3	12.5	17	19.3	20	17.9	0.5976	0.439	0.073
Taking contraception pills when missing menses	0	0	4	4.5	4	4.5	NA	NA	
Hormonal injection every three months	0	0	3	3.4	3	2.7	NA	NA	

Note: NA = Not applicable.

.10 - small effect size.

.30 - medium effect size.

.50 - large effect size.

Only 17.9 percent of the respondents knew the correct time-frame for an effective use of ECPs to prevent pregnancy recognizing the need to take the first dose within 72 hours after having unprotected sexual intercourse. Women were more aware about the correct time compared to men (19.3% vs 12.5%; p > .05). Among those who had never heard about ECPs, 68.0 percent expressed a desire to know about the effectiveness of the method and 61.3% the method of usage (Table 4).

**Table 4:** Desire to know about ECPs among those who never heard about it (n = 388) with yes answers

**Variables**	**Male**		**Female**		**Total**		**Chi-square**	**P-value**	**Effect size**
	**n = 76**	**%**	**n = 312**	**%**	**N = 388**	**%**			
Method usage	45	59.2	193	61.8	239	61.6	0.180	0.671	0.021
Effectiveness	45	59.2	219	70.2	264	68.0	3.389	0.066	0.093
Side effects	41	53.9	168	53.8	209	53.9	0.0003	0.987	0.001
Frequency of usage	8	10.5	31	9.9	39	10.1	0.0236	0.878	0.007
Where it can be purchased	4	5.2	10	3.2	14	3.6	3.372	0.066	0.097
Price	3	3.9	14	4.5	17	4.4	0.0425	0.837	0.010

Note: .10 - small effect size.

.30 - medium effect size.

.50 - large effect size.

### Attitudes towards potential emergency contraception users

When asked about the appropriate potential users for emergency contraceptive pills (among those who knew of ECPs), the respondents cited teenagers (76%); women in reproductive age (28.4%); and other women who have had unprotected intercourse (18.8%).

The respondents demonstrated support for ECPs. About 85 percent believed that ECPs should be available in Laos and most of them mentioned the importance of preventing unwanted pregnancies. Regarding availability of ECPs in Laos, women were more likely to support the availability of ECP than men (86.5% vs 81%, p < .05), which is a reflection of the fact that women expressed more interest in the use of ECPs. The respondents were asked about future use of ECPs if needed, and about two thirds of respondents (66.8%) said that they would use them. There was a significant difference between men and women in the perception of future use of ECPs. Women themselves expressed more interest in the use of ECPs in the future compared to men (69.3% vs 57%, p < .05). Among those who agreed that they would use ECPs, the reasons cited were effectiveness (60.8%), ease of use (51.5%), and lack of side effects (13.8%) (Table 5).

**Table 5:** Reasons for willingness to use or not use ECPs in future with yes answers

**Variables**	**Male**		**Female**		**Total**		**Chi-square**	**P-value**	**Effect size**
	**N = 57**	**%**	**N = 277**	**%**	**N = 334**	**%**			
**Reasons for using ECPs (n = 334)**									
Easy to use	26	45.6	146	52.7	172	51.5	0.952	0.383	0.053
Cheap price	0	0.0	13	4.7	13	3.9	2.783	0.0.095	0.091
Minimum side effects	5	8.8	41	14.8	46	13.8	1.447	0.293	0.065
High effectiveness	37	64.9	166	59.9	203	60.8	0.493	0.483	0.038
**Reasons for non-use of ECPs (n = 166)**	**Male N = 43**	**%**	**Female N = 123**	**%**	**Total N = 166**	**%**			
Do not know selling place	1	2.3	3	2.4	4	2.4	0.001	0.967	0.003
Do not know how to use	2	4.6	11	8.9	13	7.8	0.813	0.367	0.070
No one gives information	2	4.6	9	7.3	11	6.6	0.366	0.545	0.047
Concerned about health effects	36	83.7	70	56.9	106	63.8	9.22	0.002	0.244
Concerned about effectiveness	4	9.3	17	13.8	21	12.6	0.588	0.443	0.059
Concerned about the price	0	0.0	5	4.1	5	3.0	1.802	0.179	0.104

Note: Multiple responses.

.10 - small effect size.

.30 - medium effect size.

.50 - large effect size.

Those 166 subjects (33.2%) who stated that they would not use ECPs in the future, referred to concern about health effects (63.8%), concern about lack of effectiveness (12.6%), the fact that no one had told them about the ECPs (7.8%) and limited information regarding the use of the pills (6.6%) (Table 5).

When asked to identify the best way to promote knowledge regarding emergency contraceptive pills, community campaigns (39.4%) were the most widely cited followed by television (38.6%), radio (34.4%), brochures (18.2%), and posters (15.4%.)

### Factors associated with awareness of ECPs

Logistic regression was performed to identify factors associated with awareness of ECPs after controlling for confounding factors. Table 6 shows that after adjusting for the socio-demographic status of respondents, respondents with older age were significantly more likely to have heard of ECPs than their younger counterparts (OR = 2.91, 95% CI = 1.29-6.57, p = 0.010). The OR value of 2.91 suggests that while there is a statistically significant difference between younger and older age groups the effect size, that is the strength of the relationship between the two age groups is medium. Male respondents were also more likely to have heard of ECPs than female respondents (OR = 2.78, 95% CI = 1.13-6.83, p = .025), which suggests that while there is a statistically significant difference between males and females the effect size between males and females is medium.

**Table 6:** Factors associated with the awareness of ECPs among young people

**Variables**	**Had heard of ECPs (n)**	**Crude OR**	**Adjusted OR**	**95% CI**	**P-value**
Sex					.025
Female	22.0% (88)	1	1		
Male	24.0% (24)	1.11	2.78	1.13 to 6.83	
Age					
15-19 yrs	17.5%(30)	1	1		
20-25 yrs	21.1%(51)	1.25	1.75	.95 to 3.22	.070
26-30 yrs	35.6 (31)	2.60	2.91	1.29 to 6.57	.010
Marital status					
Single	20.7%(90)	1	1		
Married and other	33.3%(22)	1.96	1.22	.62 to 2.40	.549
Education					
Primary &	19.0%(4)	1	1		
Secondary	25.0%(7)	1.41	1.22	.29 to 5.03	.779
Bachelor degree & other	22.4%(101)	1.22	.98	.31 to 3.12	.982
Occupation					
Unemployed	29.0%(9)	1	1		
Student	19.9%(44)	.60	.67	.28 to 159	.366
Non student	23.8(59)	.76	.61	.25 to 1.46	.270

## Discussion

This is the first survey concerning awareness and attitudes toward ECPs among young adults in Laos. The results revealed that awareness of ECPs among this section of the public was very low (22.4%). This finding is much lower than the studies in other developing countries including Nigeria, Kenya, India, and Mexico [7,14,15]. However, it was similar to surveys in India and South Africa indicating that 23 percent of women knew about ECPs [15,16].

Moreover, 33 percent among those who had heard about ECPs recognized that ECPs were not the same as “the abortion pill”. Very few (17.9%) knew the correct time of their use (until recently 72 hours but now extended to 120 hours) [8,9]. This is likely to be due to the fact that many health providers do not know about ECPs either because they did not prescribe these drugs due to a lack of legal availability in Laos or the fact that there has been no official promotion of ECPs. This information is essential for potential users because without it, they may use the medication incorrectly or may be afraid to use it.

This study revealed that the attitudes of the small sample of young Lao people regarding ECPs were favorable. Most respondents supported the availability of ECPs within the country. About 66.8 percent would use them if needed. Similar findings have been reported by other researchers [12,17-19]. Most of the study participants in Laos were of the opinion that many women would benefit from the availability of ECPs by protecting their sexual health in reducing unintended or unwanted pregnancies and the need for abortion. Several studies have shown that making ECPs more widely available does not lead to abuse of ECPs or adversely affect regular contraceptive use [20-23] and has no adverse impact on the risk of sexually transmitted infections [22]. However, most of those studied said they would not use ECPs in the future due to concern about side effects on health, a finding from a previous study [24].

The most important sources of information on ECPs for the potential users in this study were friends, rather than health personnel and other sources such as radio, TV, magazine and other means and this is in accordance with previous studies [20,25,26]. The information on ECPs is relatively simple to provide through an informal network, and in the light of this finding, information about ECPs could be disseminated through peer education. However, informal sources of information can easily lead to misinformation; while medical and media sources are associated with more accurate information. Thus, it is essential to train peers in order to increase ECPs awareness more accurately. Particularly, there is a need to provide information on the existence of ECPs, and the availability of the same at the public and private sector.

The study shows that male young adults were more aware of ECPs than females. It could be that males are more open to talk about sex than females. The other reason might be that it is considered acceptable for men to be sexually active outside marriage which not the case for females in Laos. This finding corresponds with previous studies in Nepal [27]. Older age groups were more likely to have heard about ECPs than were those who are younger. The reason might be that they are more educated and socialize more with friends. The other reason might be that they have had more sexual experience compared to younger age groups.

Our study has several limitations. First, due to the nature of the cross-sectional design, cause and effect could not be investigated. This was a relatively small survey conducted only in the capital city of the Lao PDR. Thus, there is a need to investigate in other settings within the country where health facilities may be more constrained. A convenient sample of young adults in entertainment places was used; thus, it is not representative for all young adults in Vientiane city and hence generalizing the results of this study to other settings must be done with caution. It was anticipated through this study to get an understanding of the public perspective in order to help register the ECPs with the Food and Drug Administration (FDA) in Laos and prepare a case to introduce the ECPs within the public sector. Due to the sensitive nature of this study, the respondent’s honesty and disclosure might be constrained. However, we tried to minimize this bias by matching sex of respondents and interviewers and building a good rapport by ensuring confidentiality and privacy.

### Conclusions and recommendations

The level of knowledge about ECPs among young adults in Vientiane is relatively low and the number of misperceptions is high. Regarding attitudes towards ECPs, the majority of respondents strongly supported the availability of ECPs in the public and private sectors.

Efforts should be focused on providing health education regarding ECPs to youth and young adults both male and female, with focus on the available methods, correct timing of use, and the health effects of ECPs. It should use all major means of communications on ECPs such as peers, media (radio, television, posters and brochures), as well as delivering messages through health facilities. It would also be interesting to repeat the survey after ECPs have been more widely available in the public and private sectors for some years.

## Competing interests

The authors declare that they have no competing interests.

## Authors’ contributions

SV developed the research proposal, designed the instrument, and collected data in the field sites, analyzed and wrote the draft manuscript. KP contributed to the study design and commented on the manuscript. VH and AP assisted in the survey instrument development, analyzed data and contributed to the final version of the manuscript. All authors read and approved the final manuscript.

## Pre-publication history

The pre-publication history for this paper can be accessed here:

http://www.biomedcentral.com/1472-6874/13/14/prepub
